# Intranasal neuropeptide Y is most effective in some aspects of acute stress compared to melatonin, oxytocin and orexin

**DOI:** 10.3389/fphar.2022.1033186

**Published:** 2022-12-02

**Authors:** Eugene Zubkov, Olga Abramova, Yana Zorkina, Aleksandra Ochneva, Valeria Ushakova, Anna Morozova, Olga Gurina, Alexander Majouga, Vladimir Chekhonin

**Affiliations:** ^1^ Department of Basic and Applied Neurobiology, V. P. Serbsky National Medical Research Center for Psychiatry and Narcology, Moscow, Russia; ^2^ Mental Health Clinic No. 1 Named After N A Alexeev, Moscow, Russia; ^3^ Department of Biology, Lomonosov Moscow State University, Moscow, Russia; ^4^ Drug Delivery Systems Laboratory, D. Mendeleev University of Chemical Technology of Russia, Moscow, Russia; ^5^ Department of Medical Nanobiotechnology, Pirogov Russian National Research Medical University, Moscow, Russia

**Keywords:** stress, rats, antidepressants, melatonin, oxytocin, neuropeptide Y, orexin

## Abstract

**Objectives:** In the current study, we compared the effects of a single intranasal administration of clomipramine with effects of four neuropeptides, melatonin, oxytocin, orexin, and neuropeptide Y, to compare them in an acute stress model.

**Methods:** The anti-stress effect was evaluated in the sucrose preference and forced swimming tests. Serum corticosterone level in rats was measured to evaluate the stress response.

**Results:** Neuropeptide Y reduced immobilization time in the Porsolt test and decreased corticosterone levels, but increased the anhedonia. Orexin had no positive effect on animal behavior, but decreased corticosterone levels. Oxytocin decreased immobilization time, maintained anhedonia at the level of control, but did not affect corticosterone levels. Melatonin demonstrated no positive effects in any of the tests.

**Conclusion:** The intranasal administered neuropeptide Y could be a promising compound for the treatment of stress disorders.

## 1 Introduction

Acute stress causes alterations in the body systems, which can be either adaptive or damaging if the stress response is inadequate or excessive. An inadequate stress response triggers pathological reactions in genetically predisposed individuals and increases the risk of psychiatric disorders (Musazzi et al., 2010; Suvrathan et al., 2010). Stressful life events are well known to cause depression, anxiety disorders and post-traumatic stress disorder (PTSD) in humans (Schneiderman et al., 2005; de Abreu et al., 2020).

Nowadays, there is little data on possible treatment options to alleviate acute stress symptoms and prevent harmful long-term effects. This is why developing efficacious treatment is an urgent task (Musazzi et al., 2010). Studying the effectiveness of potential substances decreasing the acute stress effects is of high relevance (Salari, et al., 2020), since at present there are only few drugs with rapid anti-stress and anti-depressive action. Most of existing therapies show modest efficacy after several weeks of treatment in randomized clinical trials. For many stress-induced disorders, such as PTSD and depression, which contribute to the greatest burden of disease and a high mortality rate, the long wait for the treatment effect may have destructive impacts (Insel, 2012; Boaden et al., 2020). There are some drugs effective within a few hours after administration (such as ketamine and its nasal form esketamine, scopolamine, rapastinel), but they may be dangerous because of potential abuse and other risk factors (Wohleb et al., 2017; Witkin et al., 2019). An urgent challenge is to find and investigate potential effective treatment to mitigate the immediate effects of stress and its long-term negative consequences (Borbély et al., 2022).

The use of neuropeptides and neurotransmitters such as melatonin, neuropeptide Y (NPY), oxytocin, and orexin is of particular interest. In earlier studies, these compounds demonstrated some potential for treating behavioral disorders. For example, there is some evidence for the melatonin’s anti-stress effect, due to its interaction with GABAergic, serotoninergic, glutamatergic and nitrergic neurotransmitter systems, as well as modulation of the hypothalamic-pituitary-adrenal (HPA) axis (Valdés-Tovar et al., 2018). In addition, melatonin is of significant interest because of its effect on circadian rhythms, since neurochemical/hormonal imbalances due to disturbances in circadian rhythms can lead to misalignment of biological rhythms and cause psychiatric disorders (Tonon et al., 2021). NPY also demonstrated some association with the effects of acute stress. In laboratory animals, the decrease of NPY mRNA and protein in the brain were revealed in a model of PTSD. Studies by Serova, Sabban and colleagues in rodents showed that NPY administered intranasally penetrated the brain and induced pronounced direct PTSD-relieving effects. Researchers also studied the effects of oxytocin, which is known to have anti-stress effects, in the treatment of mental disorders (Abramova et al., 2020). There is reciprocal activation between the oxytocin system and the HPA, suggesting bidirectional regulation, and increased oxytocin levels are associated with lower cortisol levels (Young Kuchenbecker et al., 2021). Intranasal oxytocin therapy was previously studied for various behavioral disorders in humans and animals and demonstrated some positive results, but its therapeutic effects need to be compared with other drugs (De Cagna et al., 2019; Abramova et al., 2020). Orexin is also involved in behavioral disorders. Low peripheral and central concentrations of orexin are also common in depressed patients and people with PTSD (Strawn et al., 2010). Single prolonged stress in rats results in decreased orexin expression and the appearance of depressive-like symptoms (Han et al., 2022). We decided to focus on the study of these four compounds, because they have previously shown some effect in the therapy of behavioral disorders. They also play role in the stress response, but there are only few researches dedicated to their intranasal therapeutic effects, and even fewer investigating the chronic effects of the substances. However, the acute stress model is convenient for an initial assessment of behavioral effects. Chronic administration could be also evaluated in the future.

An important aspect of stress therapy is the route of drug delivery. In order for the drug to affect the central nervous system, it must cross the blood brain barrier (BBB). Invasive methods of overcoming the BBB include direct injection of drugs into the brain tissue. Intranasal delivery is another method of direct transport of a therapeutic agent bypassing the BBB to treat various central nervous system disorders. This method is based on the connection between the olfactory nasal lining mucosa and the circulating cerebrospinal fluid next to the olfactory bulbs (Lochhead and Thorne, 2012). Intranasal delivery of pharmaceuticals is a non-invasive, safe and promising method of treatment. Literature analysis suggests that the intranasal administration is the most advantageous way to deliver a whole set of antidepressants of different classes to the brain (Zorkina et al., 2020).

In addition, we decided to test the possible effectiveness of the tricyclic antidepressant clomipramine in acute stress. In our recent experiment we tested intranasal administration of the clomipramine in rats. It had the same effect on rat behavior as clomipramine administered intraperitoneally (according to the classical scheme) in a model of chronic stress. But importantly, the intranasal administration of clomipramine changed the metabolomic profile in the frontal cortex and hippocampus (Abramova et al., 2021). Even though clomipramine is commonly used to treat anxiety (such as with obsessive-compulsive disorder) (Xu et al., 2021), the complete effects of clomipramine on stress behavior and hormones are yet to be investigated. Additionally, clomipramine inhibits the reuptake of serotonin and norepinephrine in the brain (Balk et al., 2010). It is known that the role of noradrenaline is great in the formation of anxious behavior in animals after acute immobilization stress. Acute immobilization stress activates norepinephrine release in a number of stress-related limbic forebrain regions. Stress-induced norepinephrine release contributes to development of anxiety-like behaviors (Morilak et al., 2005). Since clomipramine also contributes to reuptake of norepinephrine, this is another argument for using it specifically for the acute stress.

In this study, we investigated the effects of a single intranasal administration of melatonin, NPY, oxytocin, orexin and clomipramine to cope with some behavioral and biochemical effects of acute stress. We used two behavioral tests for comparison, and blood corticosterone level was evaluated too, being an important indicator of the response to stress exposure.

## 2 Materials and methods

### 2.1 Animals

Experiments were performed on male Wistar rats (*n* = 64) that weighed 200–220 g. We used rats from the Nursery for Laboratory Animals (Pushchino, RAS, Moscow region) in the experiment. All animals were kept at a constant temperature (23°C) with controlled direct lighting (12/12 h) and free access to water and food. Housing conditions and all experimental procedures were set up and maintained in accordance with Directive 2010/63/EU of 22 September 2010 and approved by the local ethical committee.

### 2.2 Design of the experiment

The animals were divided into the following experimental groups: control (C; *n* = 16); acute stress (AS; *n* = 8); acute stress with clomipramine intranasal therapy (AS + Cl; *n* = 8); acute stress with melatonin intranasal therapy (AS + Ml; *n* = 8); acute stress with NPY intranasal therapy (AS + NY; *n* = 8); acute stress with orexin intranasal therapy (AS + Or; *n* = 8); acute stress with oxytocin intranasal therapy (AS + Ox; *n* = 8).

Animals were weighed before the start of the experimental procedures and were divided into groups, so that the average weight of the animals in the group was approximately the same.

**FIGURE 1:**
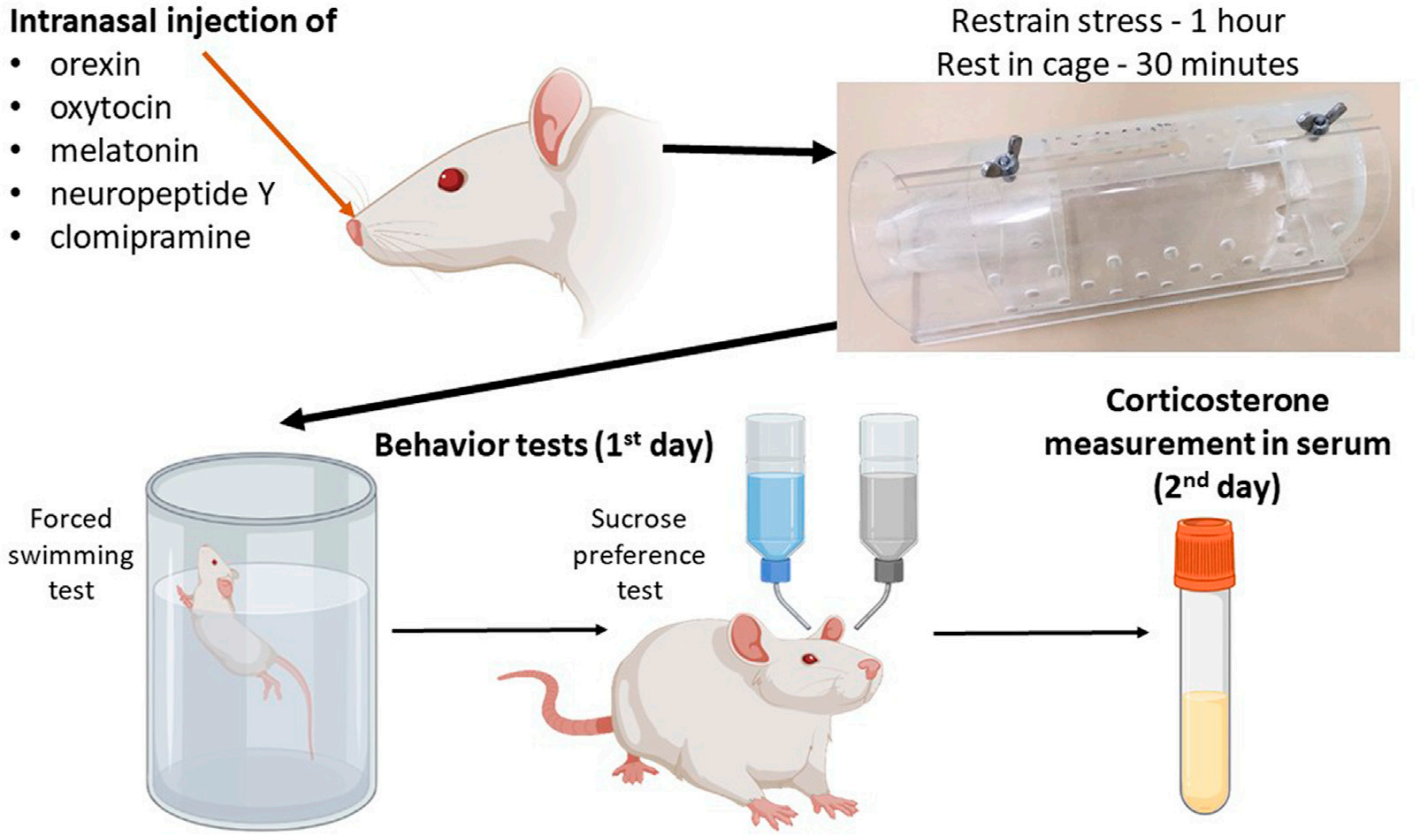
Schematic of the experiment for the drug administration groups.

The scheme of the experiment is shown in Figure 1. The control group consisted of intact rats, which were divided into two subgroups: the first subgroup (*n* = 8) was subjected to behavioral tests only, and the second subgroup (*n* = 8) had blood sampled for serum corticosterone concentration analysis to exclude the effect of test stress on corticosterone levels. Acute stress was induced by immobilizing the rats in a restrainer - plastic cone (21 сm × 7 сm × 7 cm) for 1 h. In the groups receiving therapy, neuropeptides were administered immediately after the rat was placed in the restrainer. The control group received intranasal vehicle. After the restrainer stress, the rats were placed in individual cages for 30 min to rest, then subjected immediately to a forced swimming test and then to anhedonia test. In the groups after acute stress, blood was sampled after the anhedonia test (in 24 h after the acute stress).

Blood for analysis of serum corticosterone concentration was collected after brief anesthesia followed by rapid decapitation between 12:00 and 2:00 p.m. using sterile tubes. Serum was separated immediately after blood sampling by centrifugation (3,000 rpm for 10 min) at 4°C and stored at –80°C until the analysis.

The following compounds were used in the experiment: clomipramine at a dose of 7.5 mg/kg (Clomipramine hydrochloride, 7291-5, Merck, United States ), melatonin at a dose of 50 mg/kg (M5250-5G, Merck, United States ), and peptides: NPY at a dose of 100 μg/kg, orexin A at a dose of 20 μg/kg, and oxytocin at a dose of 20 μg/kg. The substance dosing was selected in accordance with the scientific literature data. In addition, we conducted the preliminary experiment in a separate group of rats to determine the most effective dose. The low and high doses of each substance were analyzed in the forced swimming test, and the most effective dosage was selected based on the results of the test (Supplementary Figure S1). The clomipramine dose was chosen according to our previous study (Abramova et al., 2021). All peptides were synthesized by Almabion (Russia) in an amount of 14 mg. Then, the peptides were dissolved in physiological solution (NaCl 0.9%) in a volume of 1,400 μL to a concentration of 10 mg/ml. The solution was prepared immediately before use.

During intranasal administration animals were placed in plastic restrainers that were held at approximately a 45^o^ angle. When the rat’s nose was exposed to the constricted end of the restrainer, and the animal was immobilized, the intranasal injection procedure was initiated. The compounds were administered through 200 µL micropipettes using pipette dispensers. Drops were gently dripped alternately into both nostrils, with a pause for the rat to inhale a drop in the nostril before the next one was injected. The total volume of injected liquid did not exceed 30 µL. The procedure time per animal was about two minutes.

### 2.3 Anhedonia test

During the test, rats were given a free choice between two bottles for 24 h—one with a 1% sucrose solution and another—with tap water (Morozova et al., 2016). We measured the amount of fluid drunk from both bottles within 24 h and calculated the sucrose preference index (%) according to the formula: Vs./(Vs. + Vw)*100%, where Vw was the volume of pure water drunk, Vs. was the volume of 1% sucrose solution drunk. During the test bottles were swapped every six hours to eliminate place preference. Special custom made glass spouts were used for the test to prevent the spontaneous water flowing. No previous food or water deprivation was applied before the test.

### 2.4 Forced swimming test

A transparent cylindrical pool made of glass (diameter 15 cm, height 40 cm) was filled with water (24°C) to a level that prevented a rat from touching the bottom. Animals were put in the pool for 8 min. The absence of any directed movements of an animal’s head and body was considered as immobility and was kept track during the last 6 min of the test. We used the following protocol with some modifications (Bopaiah et al., 2000).

### 2.5 ELISA

Before analysis, serum samples were thawed and the amount of corticosterone was determined using an enzyme immunoassay kit (Corticosterone ELISA kit (ab108821), Abcam, United Kingdom) according to the manufacturer’s protocol. The serum was diluted 85-fold according to the protocol, and then the values obtained were calculated by increasing according to the dilution. The data obtained were recorded on a spectrophotometer (Biorad, United States ) at a wavelength of 450 nm.

### 2.6 Statistics

The RStudio and jamovi free software were used for statistical analysis and graphing. The normal distribution was verified by the Shapiro-Wilk normality test, which demonstrated a normal distribution for all data. *p*-values were calculated by using analysis of variance (ANOVA) followed by Tukey’s test for multiple comparisons. A *p*-value of <0.05 was considered statistically significant.

## 3 Results

### 3.1 Anhedonia test

**FIGURE 2:**
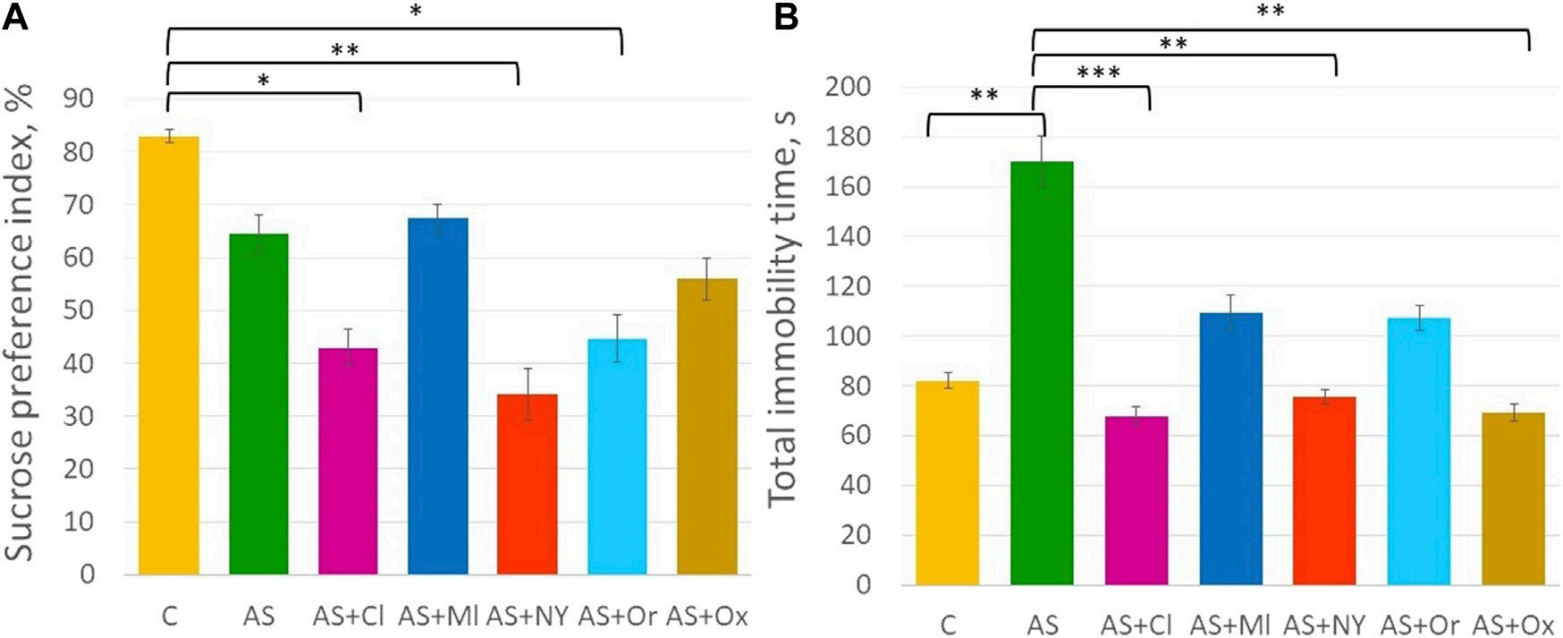
**(A)** Sucrose preference index in the anhedonia test; **(B)** Total immobility time in the forced swimming test; C—control; AS—acute stress; Cl—clomipramine; Ml—melatonin; NPY—neuropeptide Y; Or—orexin; Ox—oxytocin; *—*p* <0.05; **—*p* < 0.01; ***—*p* < 0.001.

ANOVA analysis revealed significant differences in the anhedonia test (F (6; 49) = 4.21 *p* = 0.001) (Supplementary Table S1, Figure 2A). Acute stress did not alter the sucrose preference index in the rats (*p* = 0.70). Intranasal therapy with clomipramine, NPY, and orexin after acute stress significantly decreased the sucrose preference index compared to controls (AS + Cl *p* = 0.02; AS + NY *p* = 0.002; AS + Or *p* = 0.03).

### 3.2 Forced swimming test

ANOVA indicated significant differences between groups in the forced swimming test [F (6; 49) = 4.94 *p* < 0.001] (Supplementary Table S1, Figure 2B). Acute stress significantly increased total immobility time (*p* = 0.007). Intranasal therapy with clomipramine, NPY and oxytocin significantly decreased total immobility time compared to the acute stress group (AS + Cl *p* < 0.001; AS + NY *p* = 0.003; AS + Ox *p* = 0.001). Melatonin and orexin therapy did not significantly alter total immobility time compared to the acute stress group (AS + Ml *p* = 0.14; AS + Or *p* = 0.11), but those groups did not differ from controls either (AS + Ml *p* = 0.90; AS + Or *p* = 0.93), indicating their intermediate position between control and acute stress groups.

### 3.3 Serum concentration of corticosterone

**FIGURE 3:**
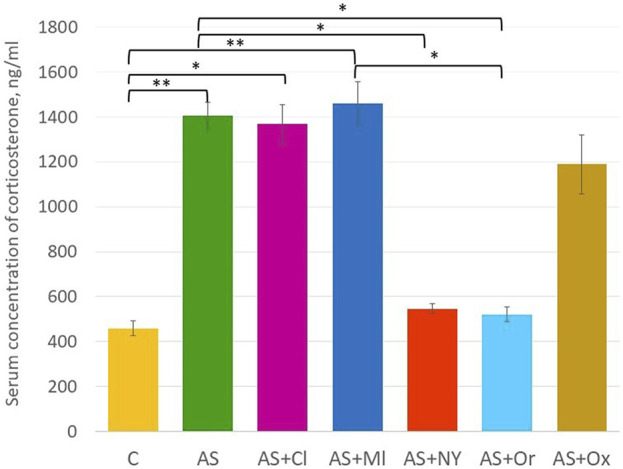
Serum concentration of corticosterone; C—control; AS—acute stress; Cl—clomipramine; Ml—melatonin; NPY—neuropeptide Y; Or—orexin; Ox—oxytocin; *—*p* <0.05; **—*p* < 0.01.

The results of serum corticosterone concentration analysis revealed significant differences between the groups [F (6; 49) = 6.18 *p* < 0.001] (Supplementary Table S1, Figure 3). Acute stress increased the serum corticosterone concentration in the rats (*p* = 0.002). NPY and orexin therapy decreased concentrations compared to the acute stress group (AS + NPY *p* = 0.02; AS + Or *p* = 0.01); clomipramine and melatonin had no effect on corticosterone levels after the acute stress (AS + Cl *p* = 1.00; AS + Ml *p* = 1.00). After oxytocin therapy, corticosterone concentrations did not differ from either the control group (*p* = 0.09) and the acute stress group (*p* = 0.89).

## 4 Discussion

The aim of our study was to compare the therapeutic effect of a single dose of intranasally administered neuropeptides and neurotransmitters such as melatonin, NPY, oxytocin, and orexin in an acute stress rat model. The antidepressant clomipramine was chosen as a comparison drug. The results of the experiment demonstrated a certain therapeutic efficacy of some neuropeptides, but there were some aspects to be discussed.

First of all, the results of the anhedonia test were confusing. Stress exposure in rats usually involves an increase of anhedonia (decreased sucrose preference index) and an increase of immobility in the forced swimming test. However, different stressing protocols can make heterogeneous effects. Our experiment included acute restraint stress, which did not affect the level of anhedonia in rats. If we analyze the work of other authors who used acute stressing, we can also see its unusual effect on anhedonia. In the Garcia-Keller et al. study rats showed an unusually increased consumption of sucrose after acute restraint stress. However, the authors noted that this phenotype was more characteristic of females than of males (Garcia-Keller et al., 2021). Orsetti et al. did not observe the formation of anhedonia in rats after acute stress induced by forced swimming in cold water (Orsetti et al., 2007). In our opinion, the sucrose intake test does not adequately reflect those behavioral changes characteristic of acute stress. Perhaps a more prolonged stressor exposure is needed to form a pronounced anhedonia. In general, many extraneous factors, such as bottle or site preference, faulty rubber stoppers, weight variance of the animals, time interval between stressor and sucrose preference test can contribute to the results of the anhedonia test, as reflected in the publications of some authors (Serova et al., 2017; Liao et al., 2021). This complicates the data interpretation and the correct assessment of the obtained effects. It may also affect the results of our study. From this point of view, the forced swimming test allows to evaluate the behavior of acute stressed animals in a more objective way. However, since the forced swimming test itself plays a role as a stressor to rodents, and is also used in some models of acute stress and depression, separate cohorts of animals for the forced swimming test and the anhedonia test conduction could be useful. In future experiments it could allow to assess the separate effect of restraint stress in animals.

Anhedonia is reported to be the most difficult symptom to treat, remaining resistant to therapy even after the elimination of other symptoms (Krystal et al., 2020). In addition, there is a risk of exacerbation of the disorder symptoms when therapy begins (Akechi et al., 2020). In our study, the compounds clomipramine, NPY, and orexin showed the reduction of the preference index in the sucrose test. We assumed that a single dose of the neuropeptides was not enough to develop a positive effect, or maybe that test was not suitable to evaluate the effectiveness of compounds in acute stress.

Although the anhedonia test did not reveal the expected result, the forced swimming test showed that the total immobility time significantly increased in rats under acute stress. At the same time, intranasal administration of clomipramine decreased the total immobility time to the level of the control animals. Therefore, in the forced swimming test, standard used for the study and testing of antidepressants, clomipramine showed its effect. It is worth noting that the forced swimming is another stressor for the rodents by itself, despite its capability for the evaluation of stress response. For this reason we address it as the limitation of our study. However, the analysis of corticosterone in the serum of rats treated with clomipramine did not reveal its decrease to the level of intact non-stressed animals. Perhaps that was due to the fact that a single administration of the antidepressant was not enough for a steady decrease in the level of the stress hormone. As for the other neuropeptides, some of the studied substances also showed their anti-stressor effect in the forced swimming test. NPY and oxytocin reduced the immobilization time to the level of control animals. At the same time, melatonin and orexin did not reveal a significant effect in reducing the total immobilization time.

Although there are literature data demonstrating anti-stress effects of melatonin, in our study it did not significantly reduce the immobilization time in rats after acute stress and had no effect on corticosterone levels. The absence of therapeutic effect of intranasal melatonin on corticosterone levels in rats can be found in the literature. However, the influence of melatonin on stress response has been shown, since the increased activation of sympathetic system and HPA axis is necessary to control melatonin production during defense reactions. The action of glucocorticoids on melatonin synthesis is controversial, with both stimulatory and inhibitory effects reported. The dual effect of corticosterone on melatonin synthesis in the pineal gland is determined by the nature of adrenoreceptor (β or β + α1) in the gland during glucocorticoids activation Fernandes et al., 2017. Despite this, melatonin has not demonstrated a significant therapeutic effect on any behavioral patterns and corticosterone as a biochemical marker of stress in the acute stress model.

According to some studies, orexin is also a promising anti-stressor compound. Orexin is associated with emotions, since the concentrations of orexin are elevated when people experience happiness, whereas a decrease of concentrations is observed during negative emotions (Blouin et al., 2013). In addition, orexin plays an important role in the sleep-wake cycle and cognitive functioning (Toor et al., 2021). Low peripheral and central concentrations of orexin were reported to be common in patients with PTSD (Strawn et al., 2010). Single prolonged stress in rats caused a decrease of orexin expression and the appearance of impaired behavior. At the same time, intracerebroventricular injection of orexin restored the disturbed behavior (Han et al., 2022). Additionally, the effect of orexin on corticosterone levels was demonstrated (Ji et al., 2019). In our study, orexin did not decrease the level of immobility in rats in the forced swimming test compared to the acute stress group, but the corticosterone level in that group turned equal to the intact animal’s level. Therefore, our study confirmed the data that orexin could positively affect at least the level of corticosterone, the biochemical marker of stress.

In animal experiments oxytocin also demonstrated its anti-stressor effect on some behavioral aspects. In our study it reduced immobility time to the level of control animals. When examining the level of corticosterone in the serum of rats, the result was intermediate between the control and the stressed group without therapy. Nevertheless, there were no statistical differences from the stress group, although literature data indicated that oxytocin could affect the HPA axis and could cause a decrease of corticosterone in rats (Petersson et al., 1999). Apparently, a single injection was not sufficient for achieving the effect. In the above study, a single injection of OX (1 mg/kg s. c.) caused a transient increase of adrenocorticotropic hormone (ACTH) and corticosterone 30 min after the injection. In contrast, the same dose decreased corticosterone, but not ACTH, 6 h after injection, but the 10 and 100 mg/kg s. c. doses of oxytocin had no effect on corticosterone concentration after 6 h. At the same time OX (1 mg/kg s. c.) administered once daily for 5 days reduced corticosterone for 10 days after the last injection. Therefore, OX appears to be able to both stimulate and suppress HPA axis activity in the short and long term, respectively. In our study, we used a relatively low dose of 20 μg/kg oxytocin; perhaps a higher dose would have been more effective.

NPY administered intranasally shows promising results in the therapy of depression in humans and in animal experiments, including stress experiments. Intranasal NPY penetrates the brain and produces a pronounced relieving effect on posttraumatic stress disorder (Sabban et al., 2015; Serova et al., 2014. (Nahvi et al., 2021). The study of plasma corticosterone level after stressor exposure showed that intranasal NPY decreased its level, in contrast to clomipramine, which did not reduce corticosterone. (Nahvi et al., 2021). Our experiments also demonstrated its anti-stressor effect in the forced swimming test along with biochemical therapeutic action. It should also be noted that usually in other studies experiments on intranasal administration of NPY were conducted under mild anesthesia, which itself produced a mild antidepressant-like effect (Weeks et al., 2013). Our experiment was performed without anesthesia and showed that some antistressor effects were caused by the direct action of NPY. In summary, NPY demonstrated the most pronounced therapeutic effect compared to the other compounds, as it was the only one effective both in the forced swimming test and in the reduction of corticosterone level.

Therefore, our study demonstrated that intranasal NPY proved to be the most effective in treating acute stress in animals. In future studies it is necessary to investigate this neuropeptide in a model of chronic stress, which could make it possible to test the antidepressant effect of NPY. As previously mentioned, stress can lead to the development of mental disorders, including depression (Schneiderman et al., 2005). It should be taken into account that various pathophysiological alterations occur during depression, such as astrocyte dysfunction (Liao et al., 2021), microglia reaction leading to stress-induced neuroinflammation (Jia et al., 2021), mitochondrial dysfunction (Bansal and Kuhad, 2016) and impaired endogenous opioidergic system (Anderson and Maes, 2020). In summary, the pathophysiology of depression is quite complex and many effects of intranasally administered neuropeptides might be mediated by different mechanisms of action. Future studies should focus on studying the relevant pathophysiological processes.

In conclusion, NPY demonstrated the most pronounced anti-stressor effect on some behavioral patterns and decreased the level of corticosterone as a biochemical marker of stress in the rats’ blood serum. The other drugs either produced only behavioral effect on immobility in the forced swimming test (oxytocin) or contributed to a decrease of corticosterone level but did not reduce the immobility time (orexin). Melatonin did not demonstrated any positive effect in the conducted tests. Among the additional conclusions of our work, it should be noted that, in our opinion, the sucrose preference test is not a suitable test for the evaluation of the acute stress response.

## Data Availability

The original contributions presented in the study are included in the article/Supplementary Material, further inquiries can be directed to the corresponding author.
