# Supplementary material for: Intranasal neuropeptide Y is most effective in some aspects of acute stress compared to melatonin, oxytocin and orexin
Source: Front Pharmacol. 2022 Dec 2;13:1033186. doi: 10.3389/fphar.2022.1033186 (PMC9755342; doi:10.3389/fphar.2022.1033186)
Supplement: Supplementary file 1 [file Table1.pdf]

Table S1. ANOVA results with Tukey's post-hoc test.

**Comparison group 1**	**Comparison group 2**	p-value		
		Serum concentration of corticosterone	Sucrose preference index in the anhedonia test	Total immobility time in the forced swimming test
Control	AS	**0.002**	0.70	**0.007**
	AS+Clomipramine	**0.01**	**0.02**	1.00
	AS+Melatonin	**0.008**	0.84	0.90
	AS+ NPY	1.00	**0.002**	1.00
	AS+Orexin	1.00	**0.03**	0.93
	AS+Oxytocin	0.09	0.27	1.00
AS	AS+Clomipramine	1.00	0.69	**< 0.001**
	AS+Melatonin	1.00	1.00	0.14
	AS+ NPY	**0.02**	0.29	**0.003**
	AS+Orexin	**0.01**	0.77	0.11
	AS+Oxytocin	0.89	1.00	**0.001**
AS+Clomipramine	AS+Melatonin	1.00	0.56	0.56
	AS+ NPY	0.10	0.99	1.00
	AS+Orexin	0.06	1.00	0.61
	AS+Oxytocin	1.00	0.96	1.00
AS+Melatonin	AS+ NPY	0.06	0.20	0.77
	AS+Orexin	**0.04**	0.64	1.00
	AS+Oxytocin	0.97	0.98	0.60
AS+NPY	AS+Orexin	1.00	0.99	0.81
	AS+Oxytocin	0.33	0.68	1.00
AS+Orexin	AS+Oxytocin	0.25	0.98	0.66
