# Supplementary material for: Intranasal neuropeptide Y is most effective in some aspects of acute stress compared to melatonin, oxytocin and orexin
Source: Front Pharmacol. 2022 Dec 2;13:1033186. doi: 10.3389/fphar.2022.1033186 (PMC9755342; doi:10.3389/fphar.2022.1033186)
Supplement: Supplementary file 2 [file Presentation1.pdf]

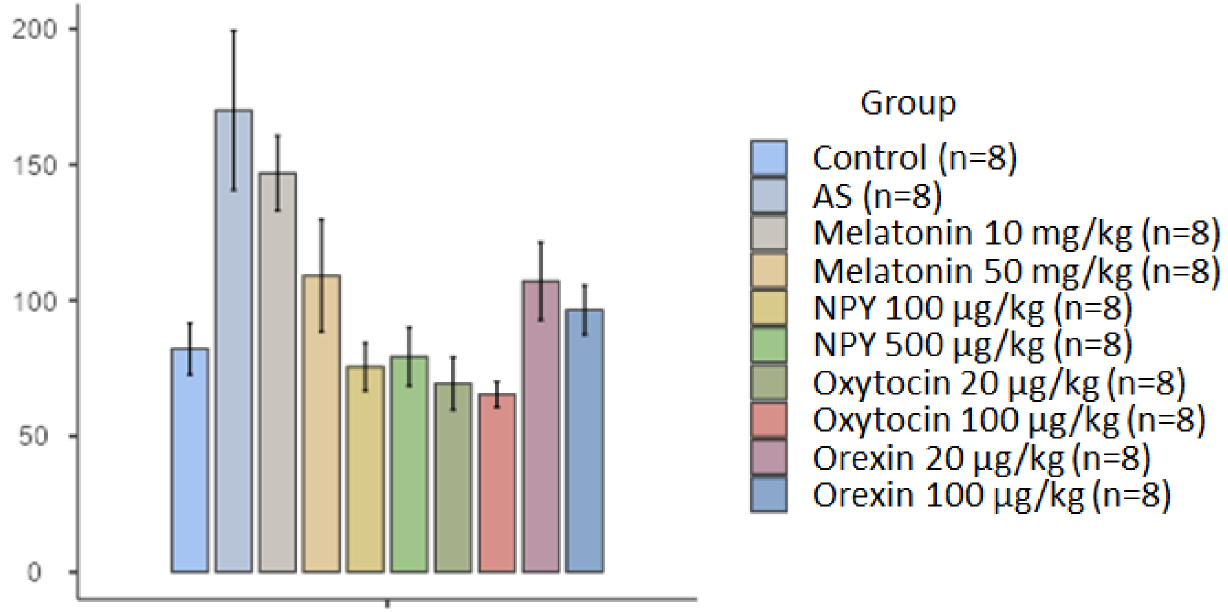

Figure S1. Results of the dose selection of the studied substances in the forced swimming test. The figure shows the mean and standard deviation; AS – acute stress;
